# Learning and the Lifespan: What’s Sex Got to Do With It?

**DOI:** 10.3389/fnins.2020.00216

**Published:** 2020-03-20

**Authors:** Amy Stave Kohtz, Cheryl A. Frye

**Affiliations:** ^1^Department of Psychology, University at Albany – State University of New York (SUNY), Albany, NY, United States; ^2^Department of Biological Sciences, University at Albany – State University of New York (SUNY), Albany, NY, United States; ^3^Center for Neuroscience Research, University at Albany – State University of New York (SUNY), Albany, NY, United States; ^4^Center for Life Sciences Research, University at Albany – State University of New York (SUNY), Albany, NY, United States

**Keywords:** cognition, aging, neurosteroids, androgens, mating

## Abstract

Engagement in sexual behavior can impact neurosteroidogenesis, in particular production of the prohormone testosterone (T) and likely its subsequent metabolism to 5α-androstane-3α-17β-Diol (3α-Diol) or aromatization to estradiol (E_2_). Androgens and their metabolites vary across the lifespan and impact many behaviors, including cognition, anxiety, and sexual behavior. Thus, we hypothesized that mating may alter cognitive performance via androstane neurosteroids in an age- and experience-dependent manner. We first investigated if exposure to mating during memory consolidation could enhance performance in the novel object recognition task (NOR). Male rats were trained in NOR and then immediately exposed to mating-relevant or control stimuli. Following a 4 h inter-trial interval (ITI), male rats were tested for object memory. Male rats that were exposed to a receptive female during the ITI had better performance in NOR. We then investigated if these effects were due to novelty associated with mating. Male rats were exposed to mating-relevant stimuli and identified as sexually responsive (SR) or sexually non-responsive (SNR) based on a median split of engagement in mating with the stimulus female. We found that a brief history (10 min session daily for five consecutive days) of sexual history substantially influenced performance in the NOR task, such that SR males had better performance in the NOR task, but only when presented with the opportunity to mate during the ITI. As T levels substantially decrease with age in male rodents, we investigated whether the effects of long-term sexual experience (10 months) influenced neurosteroids and NOR performance in mid-aged (12 months old) males. Mid-aged SR males maintain neural T; however, they have decreased neural E_2_ and decreased cognitive performance at 12 months compared to mid-aged SNR rats. In sexually experienced rats, those with better cognitive performance had greater levels of T metabolites (e.g., 3α-Diol in mated SR males, E_2_ in mid-aged SNR rats). While naïve males that were mated during the ITI had better cognitive performance, T metabolites were decreased compared to controls. These findings suggest that T metabolites, but not the prohormone, may influence learning dependent on sexual proclivity, experience, and proximate opportunity to mate.

## Highlights

- In sexually naïve rats, mating following memory acquisition can improve performance on later recognition memory testing.
- Naïve and sexually responsive (SR) male rats cognitively benefit from mating. Sexually non-responsive (SNR) males do not.
- SR rats that were mated had higher 3α-Diol across all neural targets, and higher estradiol in the cortex, compared to all other groups.
- Mid-aged SNR males have better recognition memory, and increased hippocampus and cortex estradiol, vs. SR males.
- Mid-aged SR male rats retain higher neural testosterone compared to their SNR counterparts.

## Introduction

Mating in male rats is thought to be androstane-steroid dependent and involves a number of neural substrates (Hull and Dominguez, 2007). Mating and reward increases levels of androgens, such as testosterone (T) and its 5α-reduced metabolite, 5α-androstane-3α-17β-Diol (3α-Diol), in regions that mediate reproductive and cognitive behaviors, such as the midbrain, hippocampus, and cortex (Frye et al., 2004; Edinger and Frye, 2007a, b; Frye et al., 2008; Kohtz et al., 2019). Among males, sexual performance correlates strongly to endogenous androgen concentrations (Rezek and Whalen, 1978). Similarly, castration to male rodents can decrease appetitive and consummatory sexual behavior but is restored by exogenous T (Harding and Velotta, 2011). However, there is evidence in rodents that prior reproductive experience, can ameliorate the effects of castration on engagement in mating, suggesting mating may involve learning. In fact, T actions at cognate androgen receptors (Edinger and Frye, 2007a), as well as actions of its metabolites, 3α-Diol and estradiol (E_2_), can modulate cognitive behaviors (Osborne et al., 2009; Wibowo, 2017). Thus, reproductive behaviors and cognitive performance may both be modulated by androstane steroids in male rats.

We hypothesized that increasing androstane steroid production via mating may alter cognitive performance. We first address if mating can improve cognitive performance concordant with increases in androgens in the midbrain. Then, we examined cognitive performance in sexually naïve, sexually responsive (SR) or non-responsive (non-copulatory; SNR) adult (60 days of age) male and mid-aged (12 months old) male rats to determine the effects of sexual history on cognitive performance across sexual experience, sexual proclivity, and age.

## Methods

These methods were pre-approved by The Institutional Care and Use Committee at The University at Albany – SUNY (IACUC protocol number: 10-033) and studies were conducted in compliance with ethical guidelines defined by The National Institute of Health and the Society for Neuroscience.

## Subjects

Subjects (*N* = 90) were adult male Long-Evans rats, obtained from our in-house breeding colony. The rats lived in a 12/12-h reversed light cycle (lights off at 8:00h) with unlimited access to Purina Rodent Chow and tap water in their home cages. Experimental rats were housed two (mid-aged) to four (adult naïve or sexually experienced) per cage (45 cm × 24 cm × 21cm), which contained woodchip shavings for bedding, in a temperature-controlled room (21 ± 1°C) in the Life Sciences Laboratory Animal Care Facility at The University at Albany – SUNY. All rats were trained between 10 AM and 12 PM and tested between 2 PM and 6 PM.

Rat housing was determined at weaning and did not change based for the duration of the studies, as such sexually experienced male cages housed both SR and SNR rats. Mid-aged rats were housed two per cage due to weight. While this method reduces in-cage fighting and minimizes stress associated with single-housing (SH), there is a potential confound in that cage dominance hierarchies can also indicate underlying individual differences in androstane steroids like those observed in the present manuscript. While these individual differences in neurosteroids and dominance behavior may be linked to sexual performance; social dominance was unfortunately not explicitly tested herein.

Rats that were used to determine effects of mating on cognitive performance in naïve males were randomly assigned into social exposure conditions, such that rats were non-exposed (*n* = 14), exposed to a male (*n* = 14), or exposed to a female (*n* = 15). Rats that were used to determine effects of mating on cognitive performance in sexually response (SR; *n* = 13) or sexually non-responsive (SNR; *n* = 15) males were exposed to a receptive female for 10 min per day for five consecutive days, adapted from prior reports to identify population differences in sexual performance of our colony male rats (Olivier et al., 2006). Male rats that were classified as SR engaged in mating behaviors (anogenital sniffing, mounting, intromissions, and ejaculations) for >3 of the 5 days. Male rats that were classified as SNR engaged in mating behaviors with the female stimulus rat for <2 of the 5 days. Mid-aged (12 months old) SR (*n* = 8) and mid-aged (*n* = 11) SNR rats had 10 months of variable exposure to mating, at a minimum of two mating sessions per week during which SR rats engaged in mating and SNR rats consistently did not (maintained one or less ejaculation per week for >3 months). Three days prior to testing, SR and SNR were confirmed in mid-aged rats in a single 10-min mating session with a receptive female wherein an ejaculatory series was required for SR rats and mounting behavior was absent for SNR rats.

## Behavioral Testing

### Novel Object Recognition

The novel object recognition (NOR) task is a working memory task that is cortex and hippocampus dependent (Ennaceur et al., 1997; Clark et al., 2000). This task was used as modified from previously reported methods (Ennaceur and Delacour, 1988; Frye and Lacey, 2001; Luine et al., 2003; Antunes and Biala, 2012). During training, the animal is placed in a brightly lit open field (76 cm × 57 cm × 35 cm). The open field contains two identical objects (plastic toys; Buoys, Cones, Blocks) that the animal can interact with for three minutes. Interaction was defined as time spent touching, licking, sniffing, or crawling over the objects. We used a 4 h inter-trial interval (ITI) in which the rat is returned to a single housing cage immediately (control) or following social manipulations to retain involvement of both the cortex and hippocampus (Ennaceur et al., 1997; Clark et al., 2000). Mating increases circulating T, an effect that diminishes 2 h later (Bonilla-Jaime et al., 2006; Nyby, 2008) and thus would be absent at the time of testing. During testing, one toy was replaced with a novel object, and the rat was returned to the apparatus. The percent time spent with the novel object was used as an index of cognitive performance. All rats spent at least 30 s investigating objects in both the training and testing phases.

## Standard Mating

The standard mating paradigm is a sexual behavior task in which the male rat has control over the pacing of sexual contacts and was performed similar to prior reports (Edinger and Frye, 2007b). Rats are placed in the white polycarbonate standard mating chamber (37.5 cm × 75 cm × 30 cm) for 10 min (one ejaculatory series) with a hormone-primed receptive female and the number of mounts, intromissions, and ejaculations are recorded per prior reports (Hull and Dominguez, 2007). This exposure time has been previously shown to induce neurosteroidogenesis and alter lipid profiles in female rats (Frye et al., 1996b; Frye and Bayon, 1999; Walf et al., 2011; Stuart et al., 2013).

## Tissue Collection

**FIGURE 1:**
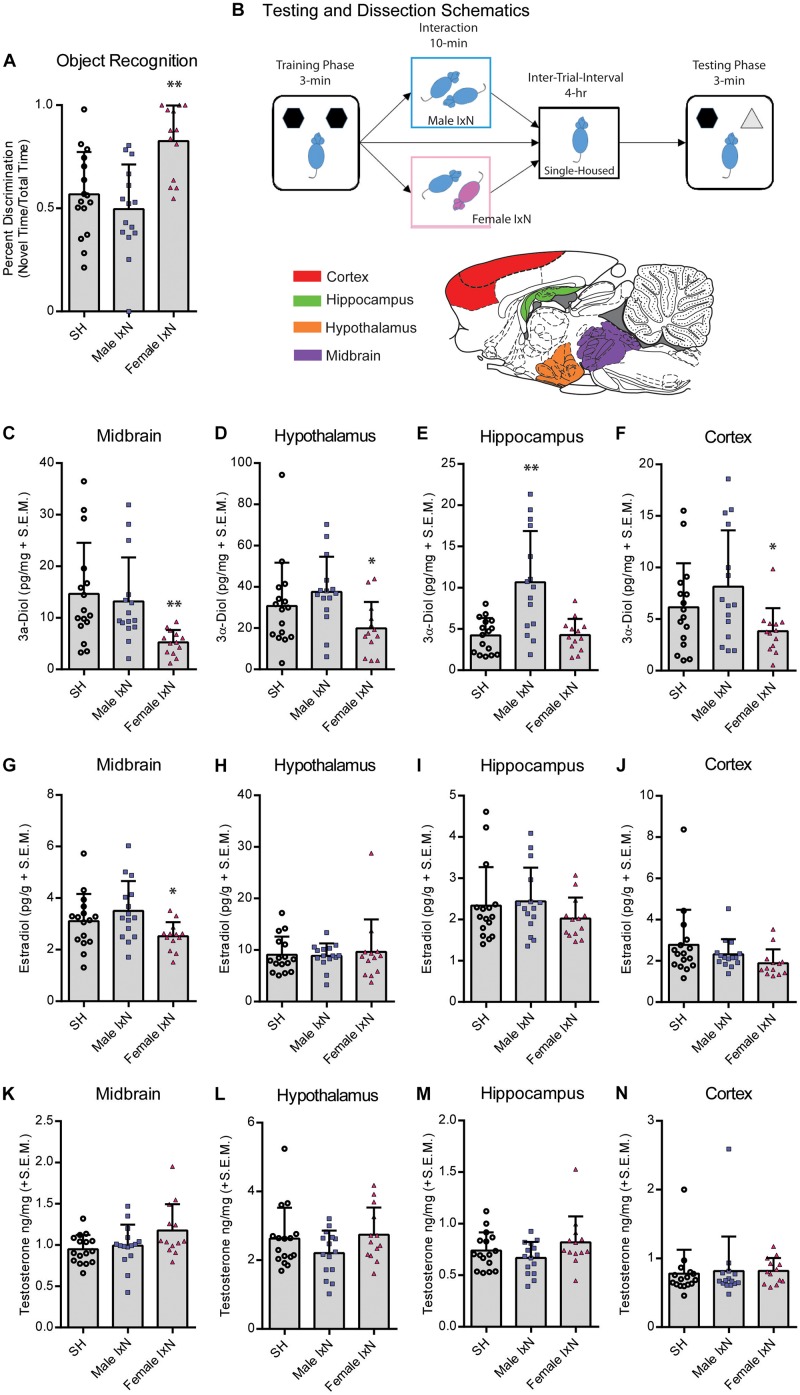
Mating during the inter-trial-interval increases cognitive performance of sexually naïve male rats. **(A)** Mating [10 min; Female interaction (IxN)] increased cognitive performance of naïve male rats compared to those that were single-housed (SH) for the entire duration of the inter-trial-interval or those that interacted briefly (10 min) with a male conspecific (Male IxN). ** indicates significantly different from both other groups *p* < 0.05. **(B)** Testing schematic of novel object recognition (NOR) and inter-trial-interval conditions for Figure 1 and the diagram of regions dissected for hormone analyses used in Figures 1C–N, 2C–N, 3C–N. **(C)** 3α-Diol (pg/mg + SEM) levels in the midbrain immediately following NOR retrieval. **(D)** 3α-Diol (pg/mg + SEM) levels in the hypothalamus immediately following NOR retrieval. **(E)** 3α-Diol (pg/mg + SEM) levels in the hippocampus immediately following NOR retrieval. **(F)** 3α-Diol (pg/mg + SEM) levels in the cortex immediately following object recognition memory retrieval. **(G)** Estradiol (pg/g + SEM) levels in the midbrain immediately following NOR retrieval. **(H)** Estradiol (pg/g + SEM) levels in the hypothalamus immediately following NOR retrieval. **(I)** Estradiol (pg/g + SEM) levels in the hippocampus immediately following NOR retrieval. **(J)** Estradiol (pg/g + SEM) levels in the cortex immediately following object recognition memory retrieval. **(K)** Testosterone (ng/mg + SEM) levels in the midbrain immediately following NOR retrieval. **(L)** Testosterone (ng/mg + SEM) levels in the hypothalamus immediately following NOR retrieval. **(M)** Testosterone (ng/mg + SEM) levels in the hippocampus immediately following NOR retrieval. **(N)** Testosterone (ng/mg + SEM) levels in the cortex immediately following NOR memory retrieval. In all figures: * indicates significantly different from rats that interacted with a male during the inter-trial-interval *p* < 0.05. ** indicates significantly different from all other groups *p* < 0.05

Immediately after testing, rats were rapidly decapitated by a well-trained individual and whole brains were collected and stored on dry ice. Whole brains were stored at -80°C for later radioimmunoassay to determine T, E_2_, and 3α-Diol levels. At the time of measurement, the hippocampus, hypothalamus, cortex, and midbrain were grossly dissected from whole brains that had been gently thawed on ice (Figure 1B).

## Radioimmunoassay

T, E_2_, and 3α-Diol were measured with radioimmunoassay techniques previously described in detail (Frye et al., 1996b; Frye and Bayon, 1999; Edinger and Frye, 2007a, b; Kohtz et al., 2019). Radioimmunoassay techniques are comparable to mass spectrometry, wherein absolute concentrations may measure differently between the two methods but overall estimates of between-subjects differences indicate similar results (Dorgan et al., 2002). Steroids were extracted from brain tissue (homogenized with a glass/Teflon homogenizer in distilled water) with diethyl ether and trace amounts of 3H 3α-Diol (purchased from New England Nuclear, Boston, MA, United States). T antibody (T3-125; Endocrine Sciences, Calabasas Hills, CA, United States) was diluted 1:2,0000 and binds between 60 and 65% of [^3^H] T (NET-387: specific activity = 51.0 Ci/mmol). The E_2_ antibody (Dr. Niswender, #244, Colorado State University, Fort Collins, CO, United States) was diluted 1:3,0000 and binds approximately 90% of [^3^H] E_2_ (NET-317: specific activity = 51.3 Ci/mmol). The antibody for 3α-Diol (X-144, Dr. P.N. Rao, Southwest Foundation for Biomedical Research, San Antonio, TX, United States) is highly specific to 3α-Diol (Rao et al., 1977). The 1:20000 dilution of this antibody binds 96% of [^3^H] 3α-Diol (NET-806: specific activity = 41.00 Ci/mmol). All standard curves were prepared in duplicate (range = 50–2000 pg). The standards were added to phosphate assay buffer, followed by addition of the appropriate antibody and [^3^H] steroid and incubated overnight at 4°C. Separation of bound and free steroid occurred by addition of dextran-coated charcoal. Following incubation with charcoal, samples were centrifuged at 3000 *g*. The supernatant was pipetted into a glass scintillation vial with scintillation cocktail. Sample tube concentrations were calculated using the logit-log method (Rodbard and Hutt, 1974), interpolation of the standards and correction for recovery. The intra- and inter-assay coefficients of variance are T = 0.09 and 0.09, 3α-Diol = 0.09 and 0.11, E_2_ = 0.09 and 0.09, respectively.

## Statistics and Experimental Design

All statistical analyses were performed in GraphPad Prism 6.0 using independent *t*-tests, or one- or two-way ANOVAs as indicated below. *Post hoc* comparisons were conducted using Bonferroni’s multiple comparisons tests. Experiment 1: Naïve male rats (∼55 days old) were trained in NOR and allowed to freely interact with a male conspecific, or receptive female conspecific, for 10-min. Following exposure to the conspecific, rats were returned to a SH unit for the remainder of the 4 h ITI. Controls remained in the SH unit for the full 4 h duration. Rats were immediately euthanized via rapid decapitation following NOR testing. One-way between-subjects ANOVAs were used to analyze the effects of the sex of the exposure conspecific on NOR performance tested 4 h later, and subsequent neurosteroid levels collected at the time of retrieval.

Experiment 2: Naïve male rats were tested for five consecutive days to determine sexual responsivity. Rats were exposed to a receptive female conspecific for 10 min each day, and engagement in mating was recorded as successfully ejaculating during the 10 min session. Rats were then distributed into SR or non-responsive groups by a criterion of 0–2 days out of 5 days of successful sessions (SNR) or 3–5 days out of 5 days of successful sessions (SR). Two-way between-subjects ANOVAs were used to compare the effects of SNR/SR and ITI condition (SH cage for 4 h, or 10 min of interaction with a sexually receptive female conspecific then SH for the remainder of the 4 h) on NOR performance.

Experiment 3: Male rats had 3–5× weekly sexual experiences for 10 months. Successful ejaculatory series were required for each week in SR mid-aged males, and a lack of intromissions were required for SNR mid-aged males. *t*-Tests were used to determine the effects of sexual responsivity at 12 months of age on cognitive performance and on neurosteroid levels following cognitive testing, as in Experiment 1.

## Results

### Mating During the Inter-Trial-Interval (ITI) Increased Cognitive Performance of Naïve Male Rats

Naïve male rats were trained for NOR, and then either single housed (SH), socially interacted with a male, or allowed to mate in a standard mating paradigm with a receptive female for 10 min. Naïve males spent an average of 53.7 ± 3.1 s total interaction time during training. In the 15 rats that socially interacted with a male conspecific there was an average of 217 ± 36 s spent interacting during the 10 min session. Among the 13 naïve rats that were matched with a female conspecific: 12 successfully mounted, 9 intromitted, and 3 performed a full ejaculatory series. Following social conditions, all rats were single housed for the remainder of the 4 h ITI (Figure 1B). Sampling of neurosteroids was taken immediately following NOR memory retrieval. Male rats that were standard mated during the ITI had increased cognitive performance on the object recognition task (*F*_2_,_41_ = 10.24, *p* < 0.05; Figure 1A), that corresponded to decreased 3α-Diol in the midbrain (*F*_2_,_41_ = 5.666, *p* < 0.05; Figure 1C), hypothalamus (*F*_2_,_41_ = 3.573, *p* < 0.05; Figure 1D), and cortex (*F*_2_,_41_ = 3.533, *p* < 0.05; Figure 1F), compared to those that socially interacted with a male, or were alone during the ITI (Figure 1). Exposure to social interaction with a male increased 3α-Diol in the hippocampus compared to standard mated or non-exposed controls (*F*_2_,_41_ = 12.74, *p* < 0.05; Figure 1E). There were no effects found on Estradiol (Figures 1G–J) or Testosterone (Figures 1K–N) in regions examined.

### Mating During the Inter-Trial-Interval Improved Performance in SR, but Not SNR Adult Male Rats

**FIGURE 2:**
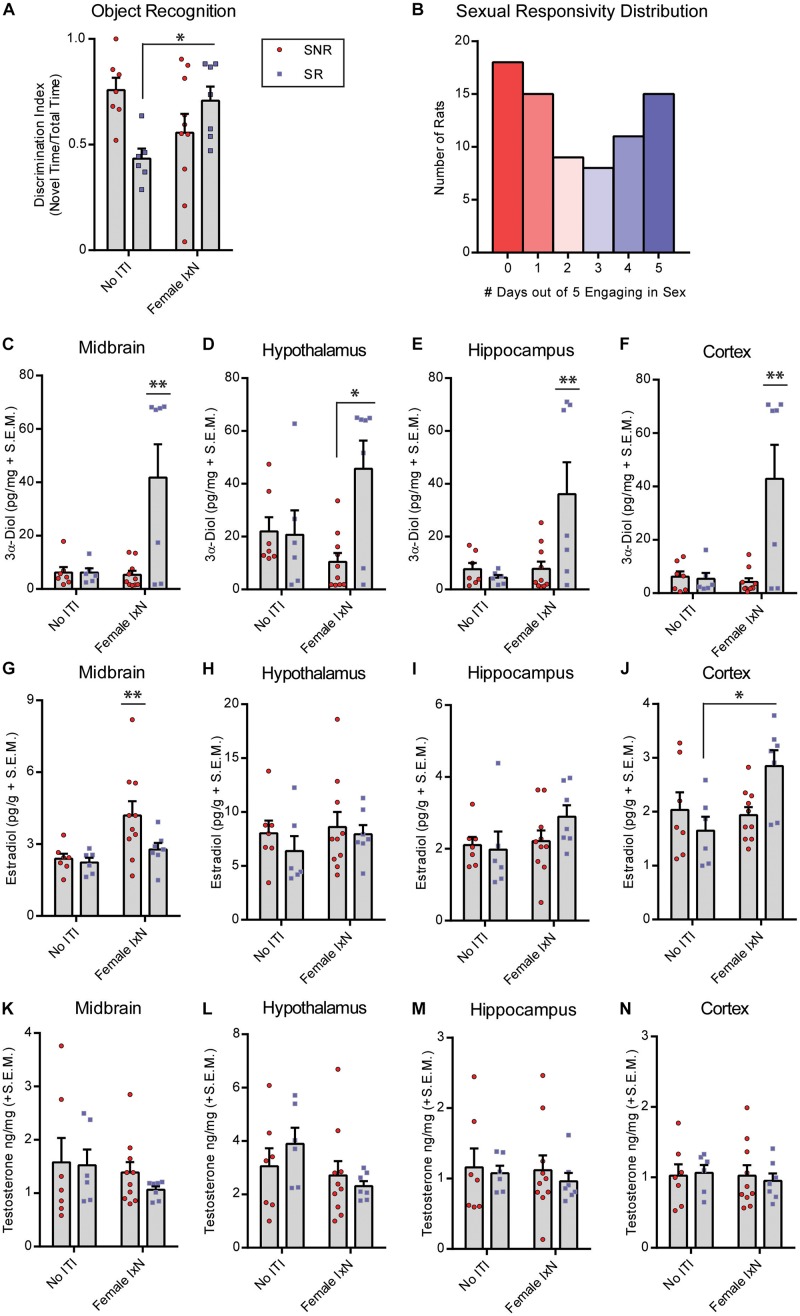
Mating during the inter-trial-interval increases cognitive performance of sexually responsive (SR), but not sexually non-responsive (SNR), young male rats. Adult male rats that were SR or SNR were ∼55–60 days of age at the time of testing. **(A)** Mating (10 min; Female IxN) increased cognitive performance of SR male rats compared to those that were single-housed (No ITI) for the entire duration of the inter-trial-interval * indicates a significant interaction between sexual responsivity and inter-trial-interval condition *p* < 0.05. **(B)** Distribution of colony rats to determine sexual responsivity by number of days engaging in sexual behavior that resulted in an ejaculatory series; a portion of these rats were used in the present study. **(C)** 3α-Diol (pg/mg + SEM) levels in the midbrain immediately following NOR retrieval. **(D)** 3α-Diol (pg/mg + SEM) levels in the hypothalamus immediately following NOR retrieval. **(E)** 3α-Diol (pg/mg + SEM) levels in the hippocampus immediately following NOR retrieval. **(F)** 3α-Diol (pg/mg + SEM) levels in the cortex immediately following object recognition memory retrieval. **(G)** Estradiol (pg/g + SEM) levels in the midbrain immediately following NOR retrieval. **(H)** Estradiol (pg/g + SEM) levels in the hypothalamus, immediately following NOR retrieval. **(I)-** Estradiol (pg/g + SEM) levels in the hippocampus immediately following object recognition memory retrieval. **(J)** Estradiol (pg/g + SEM) levels in the cortex immediately following NOR retrieval. **(K)** Testosterone (ng/mg + SEM) levels in the midbrain immediately following NOR retrieval. **(L)** Testosterone (ng/mg + SEM) levels in the hypothalamus immediately following NOR retrieval. **(M)** Testosterone (ng/mg + SEM) levels in the hippocampus immediately following NOR retrieval. **(N)** Testosterone (ng/mg + SEM) levels in the cortex immediately following NOR retrieval. In all figures * indicates significantly different from SNR rats *p* < 0.05. * indicates an interaction between sexual responsivity and inter-trial-interval condition driven by differences between the two identified groups only (*post hoc t*-tests) *p* < 0.05. ** indicates an experimental group is significantly different from all other groups *p* < 0.05.

We first determined the distribution of male rats that are prone to sexual responsivity in a population of naïve males. Rats were standard mated daily, and ejaculations were recorded as the criterion for a successful sexual encounter over 5 days. The distribution was split by 0–2 days (SNR, non-copulatory) and 3–5 days (SR) engaging in sexual behavior as described in the methods (Figure 2B). Next, we determined the effects of ITI engagement in mating or not on NOR performance in SNR and SR young males. SNR males spent an average of 49.7 ± 4.2, and SR males spent an average of 58.0 ± 5.9, seconds total interaction time during training. Of 10 SNR rats exposed to a receptive female during the ITI, 8 successfully mounted, and 6 intromitted, and 0 performed a full ejaculatory series. Of seven SR rats exposed to a receptive female during the ITI, 5/7 rats performed a full ejaculatory series. There was an interaction between sexual responsivity and ITI condition to influence performance on the NOR task (*F*_1_,_26_ = 26.41, *p* < 0.05; Figure 2A) wherein SNR males show decreased NOR memory, and SR males show increased NOR memory, when exposed to a female during the ITI. 3α-Diol levels were increased in midbrain (*F*_1_,_26_ = 17.30, *p* < 0.05; Figure 2C), hypothalamus (*F*_1_,_26_ = 16.23, *p* < 0.05; Figure 2D), hippocampus (*F*_1_,_26_ = 15.39, *p* < 0.05; Figure 2E), and cortex (*F*_1_,_26_ = 18.80, *p* < 0.05; Figure 2F) at the time of memory retrieval in SR males exposed to a female during the ITI. A multiple linear regression was calculated to predict NOR performance based on 3α-Diol across the four brain regions examined. A significant regression was found only in SR males exposed to a female during the ITI [*F*(3,26) = 26.01, *p* < 0.05], with an *R*^2^ of 0.44. NOR preference score is predicted by 57.24 + (0.4308)_*midbrain*_ + (-0.8078)_*hypothalamus*_ + (-0.0154)_*hippocampus*_ + (0.7693)_*cortex*_ 3α-Diol. Cortex E_2_ was also increased in SR males exposed to a female during the ITI (*F*_1_,_26_ = 17.28, *p* < 0.05; Figure 2J). Midbrain E_2_ was greater in SNR males compare to SR males (*F*_1_,_26_ = 17.07, *p* < 0.05; Figure 2G). There were no significant effects found in hypothalamus or hippocampus E2 (Figures 2H,I) or in T levels across brain regions measured (Figures 2K–N).

### Mid Aged-SR Males Showed Worse NOR Performance Compared to Mid Aged-SNR Males

**FIGURE 3:**
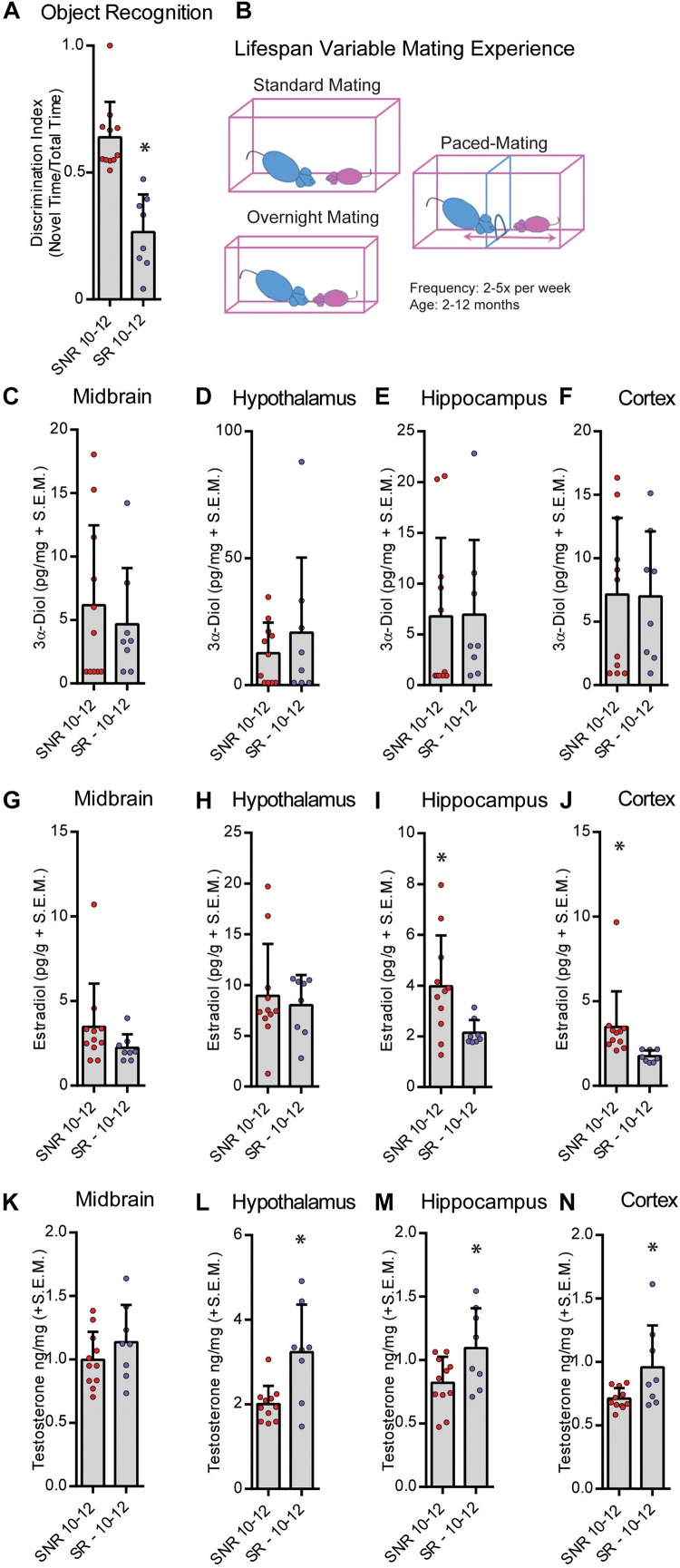
Sexual responsivity across the lifespan influences performance in the object recognition task. Aged-sexually responsive (Aged-SR) and Aged-sexually non-responsive (Aged-SNR) rats were approximately 10–12 months old at the time of testing. **(A)** Aged-SNR rats had better NOR performance compared to aged-SR rats. **(B)** Schematic depicting different mating paradigms mid-aged SR rats were exposed to weekly. Rats had between 2 and 5 days of exposure weekly for the duration of their lifespan to sexual behavior either by standard mating in which males control the pace, overnight mating for breeding, or paced-mating wherein females control the pace. **(C)** 3α-Diol (pg/mg + SEM) levels in the midbrain immediately following (NOR retrieval. **(D)** 3α-Diol (pg/mg + SEM) levels in the hypothalamus immediately following NOR retrieval. **(E)** 3α-Diol (pg/mg + SEM) levels in the hippocampus immediately following NOR retrieval. **(F)** 3α-Diol (pg/mg + SEM) levels in the cortex immediately following NOR retrieval. **(G)** Estradiol (pg/g + SEM) levels in the midbrain immediately following NOR retrieval. **(H)** Estradiol (pg/g + SEM) levels in the hypothalamus immediately following NOR retrieval. **(I)** Estradiol (pg/g + SEM) levels in the hippocampus immediately following NOR retrieval. **(J)** Estradiol (pg/g + SEM) levels in the cortex immediately following object recognition memory retrieval. **(K)** Testosterone (ng/mg + SEM) levels in the midbrain immediately following NOR retrieval. **(L)** Testosterone (ng/mg + SEM) levels in the hypothalamus immediately following NOR retrieval. **(M)** Testosterone (ng/mg + SEM) levels in the hippocampus immediately following NOR retrieval. **(N)** Testosterone (ng/mg + SEM) levels in the cortex immediately following NOR retrieval. In all figures * indicates significant difference from SNR rats (*p* < 0.05).)

We next tested the effects of sexual responsivity in mid-aged (12 months old) male rats on NOR performance. Rats were provided the opportunity to mate 2–5 times per week from 2–10 months of age prior to testing in their function as colony studs and male stimulus rats in various paradigms (Figure 3B). Responsivity and success were measured as engagement in mating similar to that of young males. Mid-aged SNR males spent an average of 52.8 ± 4.5, and mid-aged SR males spent an average of 48.1 ± 5.3, seconds total interaction time during training. In mid-aged males at 12 months, SNR males show better NOR performance compared to mid-aged SR males (*t* = 5.599, *df* = 17, *p* < 0.05; Figure 3A). SR males may in fact show an aversion to the novel object during testing (% time spent with novel object = 26.5% ± 5.26%). Mid-aged SNR males had greater hippocampus (*t* = 2.503, *df* = 17, *p* < 0.05; Figure 3I) and cortex (*T* = 2.288, *df* = 17, *p* < 0.05; Figure 3J) E_2_ levels observed at the time of memory retrieval compared to mid-aged SR males. Mid-aged SR males had greater observed T levels in hypothalamus (*t* = 3.326, *df* = 17, *p* < 0.05; Figure 3L), hippocampus (*t* = 2.305, *df* = 17, *p* < 0.05; Figure 3M), and cortex (*T* = 2.406, *df* = 17, *p* < 0.05; Figure 3N) compared to mid-aged SNR males. There were no significant effects observed in midbrain 3α-diol (Figure 3C), hypothalamus, hippocampus or cortex E2 (Figures 3D–H), or midbrain T (Figure 3K). Overall effect sizes on neurosteroid levels in this experiment were small and likely impacted by greater individual variability of neurosteroid levels in mid-aged SR rats as compared to mid-aged SNR rats.

## Discussion

Our hypothesis that mating would increase central metabolism of androgens concomitant with improved cognitive performance was partially upheld, as some contradictory effects were observed. First, NOR performance was impacted by proximate contact with a female conspecific, in a manner that depended on both prior mating experience (Naïve vs. SNR/SR), and on age (adult vs. mid-aged). We show that proximate sexual experience positively impacts cognitive performance if the subject is naïve or SR, but not if the subject is SNR. Second, sexual proclivity (SNR/SR) and prior sexual experience (naïve vs. experienced) impacted neurosteroids at the time of memory retrieval. In sexually experienced rats that had better NOR performance compared to other groups in their respective studies, hippocampal and cortical E_2_ and 3α-Diol were enhanced in adult SR rats that were exposed to a female during the ITI, whereas, hippocampal and cortical E_2_ only, were enhanced in SNR mid-aged rats (no exposure conditions). Conversely, in rats naïve to sexual experiences, mating induces increases in cognitive performance corresponded to zero neurosteroid measurements. Thus, underlying differences in proclivity may impact the effect of proximate experiences on behavior and corresponding neurosteroid levels but are independent from responses that occur to novelty.

Much evidence suggests that mating can be used as an unconditioned stimulus to elicit learned behaviors in multiple animal models. Male rats engage in mating more rapidly when presented with a CS that is paired to a female conspecific (Zamble et al., 1985). In addition, male rats exhibit a conditioned place preference (CPP) to a context previously paired with a receptive female rat, an effect dependent on the subject controlling the mating pace (Martinez and Paredes, 2001). In addition, CPP is interestingly dependent on sexual experience prior to and during the conditioning sessions; such that naïve rats condition to intromissions and ejaculations but sexually experienced rats require a full ejaculatory series to exhibit a preference (Camacho et al., 2004; Tenk et al., 2009). Similar effects have been observed in non-human primates (Snowdon et al., 2011), and extensive research on Pavlovian conditioning for mating has been performed in the Japanese quail. Mating can be used as an unconditioned stimulus to promote sign tracking behavior toward the conditioned stimulus in male Japanese quail (Burns and Domjan, 2001). In addition, male quail that learn conditioned signals for reproductive opportunities have increased reproductive success, compared to those that do not (Matthews et al., 2007). In males, mating can be used as a stimulus to produce one-trial learning (Hilliard et al., 1997). These prior reports indicate that mating can be used across species as a conditioned stimulus for Pavlovian conditioning. Our results extend these prior reports on Pavlovian conditioning to suggest that proximate sexual behavior to a learning task can induce generalized learning and is also dependent on prior (acute or lifetime) sexual experience.

Androgen receptors (ARs) and estradiol receptors (ERs) are widely, but selectively, distributed throughout the brain (Sar and Stumpf, 1981); allowing for extensive modulatory effects on behavior. ARs and ERs are expressed in the cortex and hippocampus (Lieberburg et al., 1977; Kritzer, 1997; Aubele and Kritzer, 2012). Notably, E_2_ facilitates long-term potentiation (LTP) in both cortex (Xiao et al., 2013; Galvin and Ninan, 2014) and hippocampus (Córdoba Montoya and Carrer, 1997; Smith and McMahon, 2006) whereas T facilitates long-term depression in the hippocampus (Pettorossi et al., 2013; Scarduzio et al., 2013; Di Mauro et al., 2015). In adult and mid-aged rats, cortical and/or hippocampal E_2_ was enhanced, and in mid-aged rats T was decreased, in groups that had better cognitive performance. Our data indicate increased cortex E_2_ in SR young males exposed to a female during the ITI that corresponded to better cognitive performance in nOR compared to SNR rats or SR rats that did not have female contact. In SNR mid-aged males, cortex and hippocampus E_2_ was greater than, while T was less than, SR mid-aged males. In mid-aged animals, SNR males had better cognitive performance. We hypothesize that increased cortical E_2_, and decreased T in mid-aged SNR rats only, contributed to greater facilitation of object memory retrieval likely through E_2_ facilitation of LTP.

Of interest, VTA dopamine neuron cell bodies in the midbrain extend projections to the hippocampus and cortex (Martig and Mizumori, 2011a) and express ARs, ERs, and GABA_*A*_ receptors. Reinforcement during conditioning experiments and novelty detection are tightly coupled to VTA firing (Oades et al., 1987; Martig and Mizumori, 2011a, b). 3α-Diol, a major metabolite of T, is a positive allosteric modulator of GABA_*A*_ receptors (Frye et al., 1996a; Reddy and Jian, 2010). There is some evidence that 3α-Diol is rewarding and modulates DA, as it induces a CPP when applied to nucleus accumbens shell that is abolished by 6-OHDA lesions that eliminate DA in the nucleus accumbens shell, but not core (Rosellini et al., 2001; Frye et al., 2002; Frye, 2007). In addition, DA-dependent LTP in striatum may require local synthesis of E_2_ (Tozzi et al., 2015). We hypothesize that neurosteroids, in particular T metabolites, 3α-Diol and E_2_, may tightly couple to striatal (accumbens) DA signaling. Given prior reports on the importance of DA release timing for facilitating both memory enhancement and inhibition (Grogan et al., 2015; Clos et al., 2019), 3α-Diol actions at GABA_*A*_ on DA neurons may similarly exert U-shaped effects on cognitive performance. Applying this to our model, 3α-Diol depletion (e.g., in naïve males mated during the ITI) or enhancement (e.g., in SR males mated during the ITI) may in fact facilitate object memory retrieval. Further research is required to understand the profile of androstane neurosteroids in response to mating in males, and thus our interpretation of mechanism herein is mainly speculative. While our results are consistent with findings that suggest complex androgen involvement in motivational processes, possibly through monoaminergic projection neurons or modulation of GABAergic signaling in the midbrain and limbic system, further experiments are required to address these complex interactions.

In humans, mild cognitive impairment has been associated with declining androgens during andropause (Tan et al., 2003; Fuller et al., 2007). Aging in male rats is met with substantial, albeit gradual, decline in androgen steroids concomitant with decreased cognitive performance and increased anxiety and depressive behaviors (Frye et al., 2008). Prior results indicate that administration of androgen metabolites, such as 3α-Diol may mitigate these effects (Frye et al., 2010). Interestingly, mid aged-SR males showed increased T in the hypothalamus, hippocampus, and cortex at 12 months compared to mid aged-SNR males; whereas mid aged-SNR rats had greater cortical and hippocampal E_2_. Notably, mid aged-SR rats had decreased cognitive performance at 12 months, compared to males that did not engage in mating. A facilitating role of E_2_ on cognitive performance in males, while distinct from the role of T, has been previously shown and may be particularly evident in castrated or aged males (Luine and Rodriguez, 1994; Packard et al., 1996; Bimonte et al., 2003; Gibbs, 2005). Thus, we hypothesize that these effects may be due to cortical and limbic aromatase that is limited by engagement in mating and will investigate the relationship between mating and neural steroidogenic enzyme activity in aging in future studies.

In summary, we observed changes in androgen metabolites across groups that corresponded to altered cognition and were impacted by exposure to a female (or not) occurring 4 h prior to sampling. Mating-induced T spiking is absent within 2 h (Shulman and Spritzer, 2014), suggesting the effects of our manipulations are occurring in a short window (1–2 h) following training and are not due to a prolonged presence of steroids at the time of testing. Our data expand on prior reports indicating that androstane steroids are produced in response to mating, and extend these findings to suggest that production of androstane steroids in response to mating has functional significance for mediating cognitive outcomes, albeit perhaps not in sexually naïve rats. We add that this is dependent on both sexual proclivity and proximate sexual behavior. In addition, we show that individual differences in proclivity to engage in sexual behaviors extend through mid-age, suggesting an impact on long-term cognitive performance.

## Data Availability Statement

The datasets generated for this study are available on request to the corresponding author.

## Ethics Statement

The animal study was reviewed and approved by The Institutional Care and Use Committee at The University at Albany – SUNY.

## Author Contributions

AK and CF designed all experiments, analyzed the data, and wrote the manuscript. AK performed experiments.

## Conflict of Interest

The authors declare that the research was conducted in the absence of any commercial or financial relationships that could be construed as a potential conflict of interest.
